# The effect of perinatal brachial plexus lesion on upper limb development

**DOI:** 10.1186/1471-2474-15-116

**Published:** 2014-04-02

**Authors:** Jerzy Gosk, Witold Wnukiewicz, Maciej Urban

**Affiliations:** 1 Department of Traumatology, Clinic of Traumatology and Hand Surgery, Wroclaw Medical University, ul. Borowska 213, 50-556 Wrocław, Poland

**Keywords:** Brachial plexus palsy, Limb length discrepancy, Limb girth discrepancy, Reconstruction

## Abstract

**Background:**

Deficiency in upper limb development is a sequel of the perinatal brachial plexus palsy. The purpose of this study was to evaluate the effect of brachial plexus birth lesion on upper limb development.

**Methods:**

Forty-four patients with unilateral obstetric brachial plexus palsy underwent measurements of both upper extremities. The average age at the time of evaluation was 6.8 years. Active motion was assessed using Gilbert-Raimondi, the modified MRC, and Al-Qattan scales. Paired t test was used for statistical analysis. Correlation between limb length / circumference discrepancy and age / time of surgery was assessed using Pearson correlation coefficient.

**Results:**

A decrease in the circumference and length was observed in all limbs with brachial plexus lesion. We found a statistically significant difference between degree of hand length and width decrease and its useful and useless function. We observed a statistically significant difference in measurement: forearm length, hand length and width dependent on the type of surgical procedure (neurolysis, reconstruction). We observed no correlation between age and limb length / circumference discrepancy. We also observed no correlation between time of surgery and limb length / circumference discrepancy.

**Conclusions:**

The decrease in dimensions of the affected limbs occurred predominantly during the period of early childhood. Disparities in dimensions are observed in both cases of deficiency of useful function of upper limb and cases in which functional efficiency appears.

## Background

The most common consequences of obstetric brachial plexus lesion include the neurological symptoms [1-9] and deficiency in upper limb development [10-13]. Differences in upper limb length between healthy and affected limbs are more pronounced in total brachial plexus lesions than upper lesions [11]. McDaid et al. analysed a group of 22 patients ranging from 4 to 16 years of age and concluded that discrepancies depend on lesion's location. In cases of total lesions, the shortening was greater in measurements of both whole limb length and forearm length, whilst in cases of upper brachial plexus lesions a subtler tendency to shortening of the forearm was observed [12].

The occurrence of disorders of upper limb development in the course of perinatal brachial plexus palsy is a partially unexplored phenomenon while the availability of publications on the subject is limited. The purpose of this study was to determine the limb circumference and length discrepancies in children with obstetric brachial plexus palsy who underwent primary brachial plexus exploration and to analyse the results in relation to the type of palsy, age of the patient, the timing of primary surgery, and the type of the surgical procedure.

## Methods

The study included 44 cases (30 boys, 14 girls) with obstetric brachial plexus palsy (32 cases with injury on the right side and 12 cases with injury on the left; 18 patients with upper-middle and 26 patients with total brachial plexus lesions). All children were treated surgically during infancy in the form of neurolysis (n = 28) or reconstruction (n = 16). The types of reconstruction included: direct neurorrhaphy – 6 cases, reconstruction with sural nerve grafts – 2 cases and extra-anatomical reconstruction – 8 cases (avulsion injuries). The reconstructions were performed only in cases with total brachial plexus injuries. The indication for surgical intervention was based on clinical examination and diagnostic imaging (CT myelography, MRI). The mean time between birth and surgery was 5.4 (SD 3.1) months (range 3-12 months).

The Study was approved by Wroclaw Medical University Local Bioethics Committee. Written informed consent for participation in the study was obtained from a parent.

The study group included 18 patients with upper-middle and 26 patients with total brachial plexus lesions ranging in age from 2 to 16 years (males: 2- 16 years, females: 3 -13 years). The mean age was 6.8 (SD 4.6) years (male: 6.4, female: 7.5).

### Measurements technique and scales of evaluation

The following measurements of affected and healthy limbs were taken: arm and forearm circumferences; arm, forearm and hand lengths; and hand width. The measurements of arm circumference were made at mid-arm and of forearm at one-third proximal. Length of each part of upper limb was measured in accordance to the rules given by Anand and Birch: from lateral tip of the acromion to the olecranon, from the olecranon to the tip of ulnar styloid, from the tip of the ulnar styloid to the tip of the middle finger [14]. Hand width was measured from the base of the first web space to the ulnar border of the hand [10]. Measurements were made with an orthopaedic measuring tape with preciseness to 0.1 cm.

The shoulder function was assessed using Gilbert scale [8] - Table 1. Elbow function was assessed using Gilbert and Raimondi scale [8] - Table 2. Wrist function was assessed using a modified MRC scale [15] - Table 3. Hand function was assessed using Al-Qattan scale [15] - Table 4.

**Table 1:** Gilbert’s scale for evaluation of shoulder function

Stage 0	Complete shoulder flail
Stage I	Abduction or flexion to 45°, no active external rotation
Stage II	Abduction < 90°, external rotation to neutral
Stage III	Abduction = 90°, weak external rotation
Stage IV	Abduction < 120°, incomplete external rotation
Stage V	Abduction > 120°, active external rotation

Evaluation: stage III, IV, V are functionally useful.

**Table 2:** Gilbert’s and Raimondi’s scale for evaluation of elbow function

A. Elbow flexion	Nil or some contraction	0 points
	Incomplete flexion	2 points
	Complete flexion	3 points
B. Elbow extension	No extension	0 points
	Weak extension	1 point
	Good extension	2 points
C. Extension deficit	0-30°	0 points
	30-50°	-1 point
	More than 50°	-2 points

Evaluation: grade I – poor recovery (0-1 points), grade II – average recovery (2-3 points), grade III – good recovery (4-5 points).

Grade III is functionally useful.

**Table 3:** Modified MRC scale for evaluation of wrist function

Grade 0	No contraction or flicker of contraction
Grade 1	Active movement with gravity eliminated
Grade 2	Active movement against gravity only
Grade 3	Active movement against resistance with motion reaching ≤ 1/2 normal range
Grade 4	Active movement against resistance with motion reaching > 1/2 normal range
Grade 5	Normal power and range of motion

Evaluation: grade 3 and 4 are functionally useful both in flexion and extension (F/E).

**Table 4:** Al-Qattan’s scale for evaluation of hand motor function

Grade 0	Useless hand	Complete paralysis or slight finger motion of no use, useless thumb
Grade 1	Poor function	Only very weak grip possible
Grade 2	Fair function	There is some active flexion and/or extension of the fingers and some thumb mobility but the hand posture is intrinsic minus
Grade 3	Good function	Some as 2 but there is no intrinsic minus posture (intrinsic balance)
Grade 4	Excellent function	Near normal active finger flexion/extension and thumb mobility, with some active intrinsic function
Grade 5	Normal function	

Evaluation: grade 3 and 4 are functionally useful.

### Statistical analysis

We carried out statistical analysis comparing all parameters both on sick and healthy side in relation to the type of palsy, and gender. We also investigated the relationship of the discrepancy of arm circumference to the recovery of useful shoulder/elbow functions. We also investigated the relationship of the discrepancy of forearm circumference to the recovery of useful wrist function; and the relationship of the discrepancy of hand dimension to the recovery of useful hand function. Finally, we investigated if the type of surgery (neurolysis vs reconstruction), and time of surgery (group A – children operated within 3 months of life, group B – children operated between 4 and 6 months, group C – children operated between 7 and 12 months) had an effect on the discrepancies of various parameters.

For statistical analysis paired t test was used for paired variables. Pearson correlation coefficient was used for estimation of the correlation between age / time of surgery and all the measures of sick limb. Statistical analysis was performed using Statistica 8.0. Statistical significance was accepted as p < 0.05.

## Results

The results are presented in Tables 5, 6, 7, 8, 9, 10 and 11.

**Table 5:** The comparison of healthy and sick limbs circumferences and lengths between upper-middle and total lesions

**Clinical view of lesion**	**ACS**^**a**^	**FCS**^**b**^	**ALS**^**c**^	**FLS**^**d**^	**HLS**^**e**^	**HWS**^**f**^
	**(%)**	**(%)**	**(%)**	**(%)**	**(%)**	**(%)**
Upper-middle	95	96	97	97	95	96
(UM)	(SD 3.6)^*^	(SD 2.4)^*^	(SD 3.2)^*^	(SD 4.2)^*^	(SD 3.4)^*^	(SD 6.2)^*^
n = 18						
Total	94	92	94	91	88	88
(T)	(SD 7.9)^*^	(SD 6.4)^*^	(SD 2.6)^*^	(SD 5.3)^*^	(SD 6.1)^*^	(SD 8.3)^*^
n = 26						
Statistical significance	ACS^a^	FCS^b^	ALS^c^	FLS^d^	HLS^e^	HWS^f^
UM / T						
p =	NS	NS	0.012	0.006	0.005	0.017

^a^- arm circumference on sick side.

^b^- forearm circumference on sick side.

^c^- arm length on sick side.

^d^- forearm length on sick side.

^e^- hand length on sick side.

^f^- hand width on sick side.

^*^- average dimension on sick side expressed in % in relation to healthy side (100%).

**Table 6:** The comparison of limb circumferences and lengths between genders and statistical significance analysis of parameters

**Gender**	**ACS**^**a**^	**FCS**^**b**^	**ALS**^**c**^	**FLS**^**d**^	**HLS**^**e**^	**HWS**^**f**^
	**(%)**	**(%)**	**(%)**	**(%)**	**(%)**	**(%)**
Male	93	93	95	94	92	91
(M)	(SD 7.4)^*^	(SD 6.0)^*^	(SD 3.0)^*^	(SD 6.4)^*^	(SD 6.4)^*^	(SD 8.4)^*^
n = 30						
Female	97	95	95	92	91	91
(F)	(SD 2.7)^*^	(SD 4.1)^*^	(SD 4.1)^*^	(SD 4.2)^*^	(SD 6.0)^*^	(SD 9.6)^*^
n = 14						
Statistical significance	ACS^a^	FCS^b^	ALS^c^	FLS^d^	HLS^e^	HWS^f^
M / F						
p =	NS	NS	NS	NS	NS	NS

^a^- arm circumference on sick side.

^b^- forearm circumference on sick side.

^c^- arm length on sick side.

^d^- forearm length on sick side.

^e^- hand length on sick side.

^f^- hand width on sick side.

^*^- average dimension on sick side expressed in % in relation to healthy side (100%).

**Table 7:** The comparison of arm circumference and length decrease level on sick side with useful and useless shoulder function

**Shoulder function**	**ACS**^**a**^	**Statistical significance**
	**(%)**	**UF / UL**
Useful (UF)	95 (SD 3.6)^*^	p = NS
n = 38		
Useless (UL)	94 (SD 6.7)^*^	
n = 6		
Shoulder function	ALS^b^	Statistical significance
	(%)	UF / UL
Useful (UF)	95 (SD 3.4)^*^	p = NS
n = 38		
Useless (UL)	95 (SD 1.8)^*^	
n = 6		

^a^- arm circumference on sick side.

^b^- arm length on sick side.

^*^- average dimension on sick side expressed in % in relation to healthy side (100%).

**Table 8:** The comparison of the arm circumferences decrease level on sick side with useful and useless elbow function

**Elbow function**	**ACS**^**a**^	**Statistical significance**
	**(%)**	**UF / UL**
Useful (UF)	96 (SD 3.2)^*^	p = NS
n = 28		
Useless (UL)	92 (SD 9.7)^*^	
n = 16		

^a^- arm circumference on sick side.

^*^- average dimension on sick side expressed in % in relation to healthy side (100%).

**Table 9:** The comparison of forearm circumferences decrease level on sick side with useful and useless wrist function

**Wrist function**	**FCS**^**a**^	**Statistical significance**
	**(%)**	**UF / UL**
Useful (UF)	95 (SD 3.4)^*^	p = NS
n = 30		
Useless (UL)	91 (SD 7.4)^*^	
n = 14		

^a^- forearm circumference on sick side.

^*^- average dimension on sick side expressed in % in relation to healthy side (100%).

**Table 10:** The comparison of limb circumferences and lengths between groups dependent on type of surgery

**Type of surgical procedure**	**ACS**^**a**^	**FCS**^**b**^	**ALS**^**c**^	**FLS**^**d**^	**HLS**^**e**^	**HWS**^**f**^
	**(%)**	**(%)**	**(%)**	**(%)**	**(%)**	**(%)**
Neurolysis (N)	95	95	95	96	94	95
n = 28	(SD 3.6)^*^	(SD 4.0)^*^	(SD 4.0)^*^	(SD 5.6)^*^	(SD 5.5)^*^	(SD 6.2)^*^
Reconstruction (R)	93	92	95	89	87	85
n = 16	(SD 9.4)^*^	(SD 7.0)^*^	(SD 1.5)^*^	(SD 3.0)^*^	(SD 5.3)^*^	(SD 7.7)^*^
Statistical significance	ACS^a^	FCS^b^	ALS^c^	FLS^d^	HLS^e^	HWS^f^
N / R						
p =	NS	NS	NS	0.000	0.002	0.000

^a^- arm circumference on sick side.

^b^ forearm circumference on sick side.

^c^- arm length on sick side.

^d^- forearm length on sick side.

^e^- hand length on sick side.

^f^- hand width on sick side.

^*^- average dimension on sick side expressed in % in relation to healthy side (100%).

**Table 11:** The correlation between time of surgical procedure and the degree of the underdevelopment of upper extremity

**Examited parameters**	**r =**	**p =**
TS^*^ / ACS^a^ (%)	0.074630	0.630191
TS^*^ / FCS^b^ (%)	0.295188	0.051742
TS^*^ / ALS^c^ (%)	-0.212970	0.165138
TS^*^ / FLS^d^ (%)	-0.366972	0.134134
TS^*^ / HLS^e^ (%)	-0.273343	0.272419
TS^*^ / HWS^f^ (%)	0.324802	0.105456

^a^- arm circumference on sick side (in % in relation to healthy side).

^b^- forearm circumference on sick side (in % in relation to healthy side).

^c^- arm length on sick side (in % in relation to healthy side).

^d^- forearm length on sick side (in % in relation to healthy side).

^e^- hand length on sick side (in % in relation to healthy side).

^f^- hand width on sick side (in % in relation to healthy side).

^*^- time of surgery.

**Figure 1:**
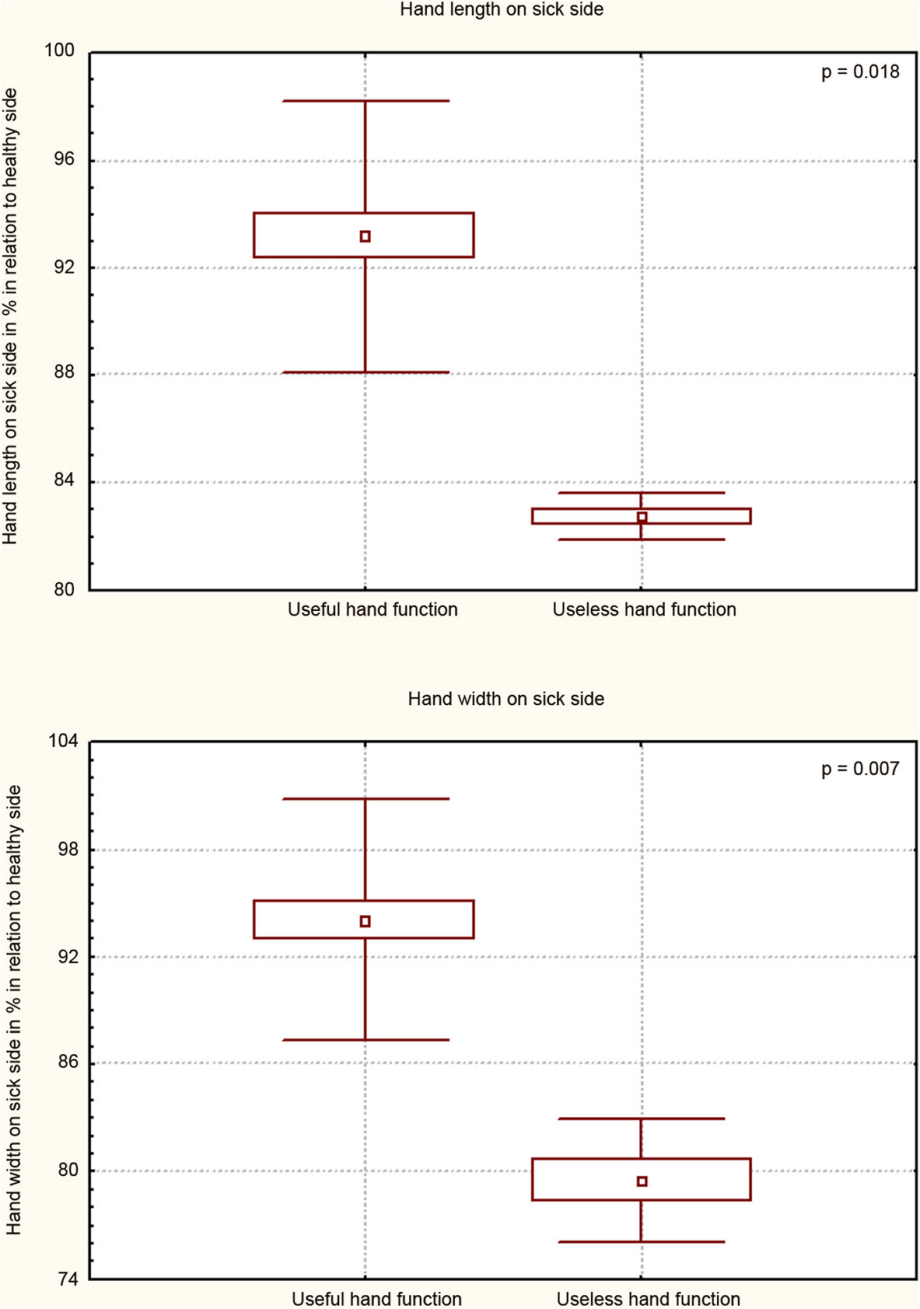
The comparison of hand dimensions decrease level on sick side with its useful and useless function.

Statistical analysis of limb measurements’ results showed a decrease of all examined circumferences and lengths on the side with brachial plexus lesion, when compared to the health side - Table 5. We observed no statistical differences between genders in all examined parameters as shown in Table 6. Furthermore, we did not observe a statistically significant difference in the degrees of decrease of arm circumference and length on sick side neither in useful nor in useless shoulder function - Table 7. We did not observe a statistically significant difference in the degrees of decrease of arm circumference on sick side neither in useful nor in useless elbow function - Table 8. Analogous observation was made in regards to the size of deficit of forearm circumference and wrist function - Table 9. We found a statistically significant difference between degree of hand length and width decrease and its useful and useless function – Figure 1. We observed a statistically significant difference in measurement: forearm length, hand length and width dependent on the type of surgical procedure (neurolysis, reconstruction) - Table 10. We observed no correlation between time of surgical procedure and the degree of the underdevelopment of upper extremity – Table 11.

## Discussion

With the incidence range of 0.5-5%, the perinatal brachial plexus palsy remains a concerning and challenging complication, despite the advances in obstetrics [10,12,13]. The majority of cases achieve a satisfactory recovery without surgical intervention [8,13] and, in particular, in cases of upper injuries of brachial plexus [5,6]. However, in total palsies, the spontaneous reinnervation may be significantly limited [6,8-10,13]. In severe cases of perinatal brachial plexus palsy, where no functional recovery is observed during first months of life, a surgical intervention is indicated [6,8,9,13]. In our study, which aimed to assess the effect of perinatal brachial plexus lesion on upper limb development, we examined a group of 44 patients of 2 to 16 years of age surgically treated during infancy.

We found, by comparing the examined dimensions between groups of upper-middle and total lesions, a statistically significant difference in the degree of decrease of arm, forearm and hand length as well as hand width. Deficit of these dimensions was shown to be more significant in total brachial plexus palsy – Table 5. As such, our results partially differ from McDaid’s observations, as he did not find differences in size of arm abbreviation despite statistically significant differences between deficit of forearm length in groups with upper and total lesions [12]. However, the most surprising observation was the decrease of hand dimensions in the group of upper-middle lesions – Table 5.

Our observations included in Tables 7, 8 and 9 reaffirm the results of Bae and co., who found no correlation between the degree of reduction of the upper limb dimensions and its motor function. They also concluded that the degree of difference should not be utilized for the purpose of estimation of upper limb impairment [10]. However, in terms of hand measurements, we found a statistically significant difference between degree of hand length and width decrease and its useful and useless function – Figure 1. This substantiates Terzis and Kokkalis’ research, in which they noted a significant correlation between upper limb length discrepancies and upper limb motions [13].

In our material we did not find a correlation between the time of the surgical procedure and the degree of the underdevelopment of upper extremity – Table 11. The correlation was found neither in the whole examined group nor in the particular types of injuries (upper-middle injuries, total injuries). We also did not observe any statistically significant differences between degree of decrease of upper extremity dimensions in particular age groups that indicated time of the surgical procedure (Groups A, B, C). This was the case for both total injuries, and upper-middle injuries. Our findings differ from the results published by Terzis and Kokkalis [13]. It might be ascribed to the difference between clinical materials and, more specifically to difference between the time of the operation and percent of total injuries. In our material the mean time between birth and surgery was 5.4 months (range 3-12 months) and the proportion of total injuries was 59%. In Terzis and Kokkalis’ research these values were 26.4 months (range 2-108 months) and 78% respectively [13]. The time between injury and microsurgical treatment is an important yet not the only factor determining the final outcome of surgery. It is also important to highlight that positive results can be achieved in children treated microsurgically in age 3, 6 or 9 months of life. In children above the age of 18 months microsurgical treatment becomes less advantageous and tendon transfers appear to be the method of choice [1,2,5-9].

While comparing the degree of decrease of upper extremity dimensions we observed that, depending on the type of surgical procedure (neurolysis, reconstruction), there is a statistically significant difference in measurement: forearm length, hand length and width – Table 10. The degree of the underdevelopment was greater in patients who underwent a microsurgical reconstructions (direct neurorrhaphy, reconstruction with sural nerve grafts, extraanatomical reconstruction). These procedures were carried out only in the group of total injuries (26 cases: 16 reconstructions, 10 neurolysis). In cases of upper-middle injuries (18 cases) with initially normal hand function only neurolysis was performed.

Furthermore, we did not find a correlation between degree of circumferences and lengths deficit on sick side and age of examined patients who ranged from 2 to 16 years of age. McDaid’s observations of a group of patients aged between 4 and 16 years (average age 8.6 years) were convergent [12]. On the basis of clinical material, which included 48 patients aged between 1 and 169 months (average age 47 months). Bae and co. drew similar conclusion and found no correlation between age and degree of decrease of examined dimensions of upper limb on injured side [10]. Therefore, we have come to the conclusion that disparities between upper limbs develop in early childhood and do not increase with age. The reason of this disorder is not fully explained [10,12,13]. The differences between upper limbs in children with obstetrical brachial plexus palsy may be generated by the incorrect function of paralysed muscles of upper limb as well as deficiency in correctly directed mechanical loads in the early period of bone-joint system development [12]. The reduction of the mechanical stresses is critical to longitudinal growth of the long bones [13]. Later deformation and stimulation of growth plates may be responsible for its premature closing [16].

Perinatal brachial plexus lesion radically affects the development of upper limb. Finally, we observed a decrease of upper limb dimension in comparison with healthy side regardless of revival of motor function. Therefore, it is important to emphasize that perinatal brachial plexus lesion poses serious challenges to parents as well as to the child itself in its later stages of life. Birth of a child with perinatal brachial plexus palsy causes parents a considerable amount of stress amplified by realisation that the child was perfectly healthy up to the point of labour [17]. Their ordeal is often prolonged by uncertain prognosis, in particular because varying degrees of neural tissue lesion might lead to similar clinical symptoms. Only a prolonged clinical observation allows for differentiation between cases of perinatal brachial plexus palsy with good prognosis and injuries where surgical treatment is necessary [1,2,5-9]. With time, upper limb dysfunction comes to pose a major problem to a child itself [18]. For many young patients such visible disability is tantamount to numerous developmental and behavioural problems aggravated in cases of severe brachial plexus lesion [18].

There is a notable scarcity of research and relevant literature on the subject of disorders of upper limb development in perinatal brachial plexus palsy. In the last ten years, only three articles were published on the subject in English language journals [10,12,13]. Each of these studies have had a retrospective nature and analysed homogenous group of patients. McDaid et al. focus on 22 cases of children who underwent tendon transfers but not microsurgical reconstructions [12]. Bae et al. analysed 48 cases treated conservatively [10]. Finally, Terzis and Kokkalis’ examine cases of 54 children who underwent microsurgical treatment [13]. The last-mentioned authors do not compare their results with a control group of children (not treated surgically). This is also the case in this work as the great majority of children admitted to our clinic require surgical procedures.

Our work aims at expanding the knowledge on the disorders of upper limb development in perinatal brachial plexus palsy and a better understanding of the phenomena. However, since the available literature offers distinctive, and sometimes contrasting observations, we point out to a necessity for a multicentre researches of prospective character.

## Conclusions

The decrease in dimensions of the affected limbs occurred predominantly during the period of early childhood. Disparities in dimensions are observed in both cases of deficiency of useful function of upper limb and cases in which functional efficiency appears.

## Competing interests

The authors declare that they have no competing interests’.

## Authors’ contributions

WW and MU collected and analyzed the clinical data with review of the literature, and participated in the design of the study. JG conceived of the study, participated in the design of the study, performed the statistical analysis, wrote the manuscript. All authors read and approved the final manuscript.

## Pre-publication history

The pre-publication history for this paper can be accessed here:

http://www.biomedcentral.com/1471-2474/15/116/prepub
